# Identification of Novel Androgen-Regulated Pathways and mRNA Isoforms through Genome-Wide Exon-Specific Profiling of the LNCaP Transcriptome

**DOI:** 10.1371/journal.pone.0029088

**Published:** 2011-12-14

**Authors:** Prabhakar Rajan, Caroline Dalgliesh, Phillippa J. Carling, Thomas Buist, Chaolin Zhang, Sushma N. Grellscheid, Kelly Armstrong, Jacqueline Stockley, Cedric Simillion, Luke Gaughan, Gabriela Kalna, Michael Q. Zhang, Craig N. Robson, Hing Y. Leung, David J. Elliott

**Affiliations:** 1 Institute of Human Genetics, Newcastle University, Newcastle-upon-Tyne, United Kingdom; 2 Beatson Institute for Cancer Research, Glasgow, United Kingdom; 3 Institute of Cancer Sciences, University of Glasgow, Glasgow, United Kingdom; 4 Cold Spring Harbor Laboratory, Cold Spring Harbor, New York, United States of America; 5 Northern Institute for Cancer Research, Newcastle University, Newcastle-upon-Tyne, United Kingdom; 6 Institute for Cell and Molecular Biosciences, Newcastle University, Newcastle-upon-Tyne, United Kingdom; The Chinese University of Hong Kong, Hong Kong

## Abstract

Androgens drive the onset and progression of prostate cancer (PCa) by modulating androgen receptor (AR) transcriptional activity. Although several microarray-based studies have identified androgen-regulated genes, here we identify in-parallel global androgen-dependent changes in both gene and alternative mRNA isoform expression by exon-level analyses of the LNCaP transcriptome. While genome-wide gene expression changes correlated well with previously-published studies, we additionally uncovered a subset of 226 novel androgen-regulated genes. Gene expression pathway analysis of this subset revealed gene clusters associated with, and including the tyrosine kinase *LYN*, as well as components of the mTOR (mammalian target of rapamycin) pathway, which is commonly dysregulated in cancer. We also identified 1279 putative androgen-regulated alternative events, of which 325 (∼25%) mapped to known alternative splicing events or alternative first/last exons. We selected 30 androgen-dependent alternative events for RT-PCR validation, including mRNAs derived from genes encoding tumour suppressors and cell cycle regulators. Of seven positively-validating events (∼23%), five events involved transcripts derived from alternative promoters of known AR gene targets. In particular, we found a novel androgen-dependent mRNA isoform derived from an alternative internal promoter within the *TSC2* tumour suppressor gene, which is predicted to encode a protein lacking an interaction domain required for mTOR inhibition. We confirmed that expression of this alternative TSC2 mRNA isoform was directly regulated by androgens, and chromatin immunoprecipitation indicated recruitment of AR to the alternative promoter region at early timepoints following androgen stimulation, which correlated with expression of alternative transcripts. Together, our data suggest that alternative mRNA isoform expression might mediate the cellular response to androgens, and may have roles in clinical PCa.

## Introduction

The growth, development and function of the prostate gland are dependent on androgens, which affect gene expression via the androgen receptor (AR), a steroid hormone receptor transcription factor. AR transcriptional activity also drives the onset and progression of prostate cancer (PCa) [1], the second leading cause of male cancer-related deaths with ∼899,000 men diagnosed worldwide each year [2]. Cognate ligand-activated AR is thought to exert its transcriptional effects via direct or indirect binding to DNA sequences termed androgen response elements (AREs) [3], [4]. Effective first and second line therapies for advanced PCa inhibit endogenous androgen production and AR function [5], highlighting the critical importance of AR signalling and downstream gene targets in PCa biology.

Alternative mRNA isoforms with different exon contents can be generated through variable alternative splicing patterns, and selection of alternative transcriptional initiation and termination sites. Using these mechanisms, mRNAs with different exon combinations are transcribed from most (∼70 to 90%) human genes [6], [7], [8], and can encode proteins with different sub-cellular localisations and functions. In addition to transcriptional effects, androgens and other steroid hormones are implicated in the post-transcriptional regulation of gene expression [9], [10], [11], [12], [13]. AR has also been shown to modulate expression of mRNA isoforms regulated by alternative pre-mRNA splicing and promoter selection [14], [15]. In PCa, there is emerging evidence to suggest that expression of specific splice isoforms derived from cancer-relevant genes may underlie tumour biology and contribute to clinical disease progression [15].

Several genome-wide studies have successfully identified androgen-regulated transcriptional events using PCa models [16], [17], [18], [19], [20], [21], [22], but have been unable to discern between alternative mRNA species due microarray design or platform-specific analysis issues. Newer splicing-specific microarrays and high throughput sequencing (mRNA-Seq) approaches theoretically allow identification of all mRNA isoforms as well as determination of gene expression in PCa [15]. An mRNA-Seq-based analysis of the androgen-responsive LNCaP cell transcriptome [22] was able to detect important differences in gene expression, but did not achieve the required tag density to identify quantitative changes in mRNA isoform abundance.

In this study, our objective was to identify androgen-regulated alternative mRNA isoforms generated by alternative splicing and from alternative transcriptional initiation sites due to promoter selection. Screening for gene expression changes at exon-level resolution also enabled us to identify novel AR-regulated genes and associated pathways. In particular, we identified clusters of novel androgen-regulated genes associated with, and including the tyrosine kinase LYN, and a novel mRNA isoform derived from the Tuberous Sclerosis Complex *TSC2* tumour suppressor gene under the control of an AR-dependent alternative internal promoter. This novel *TSC2* mRNA contains the reading frame for only the carboxy (C)-terminal region of the tuberin protein, and was predicted to lack an interaction domain required for heterodimerisation with hamartin to enable inhibition of the mammalian target of rapamycin (mTOR) pathway, which is commonly dysregulated in cancer.

## Results

### Exon-specific analyses of the androgen-responsive LNCaP transcriptome identifies novel androgen-regulated genes and mRNA isoforms

To monitor the global response of the LNCaP transcriptome to androgen stimulation at single exon-level resolution, total RNA was extracted from LNCaP cells grown in steroid-deplete medium (n = 4) and following 24 hours of treatment (n = 4) with 10 nM synthetic androgen analogue R1881. The 24 hour time point is consistent with a recently published kinetic analysis of androgen-regulated gene expression, which reported a robust genome-wide transcriptional response to androgen stimulation after 24 hours [17]. To confirm an AR-dependent transcriptional response, we used qPCR to demonstrate up-regulation of *KLK3* gene expression in each of the treatment samples as compared with controls (Figure S1). Using the Human Exon 1.0 ST Array, we profiled expression of more than one million exons in a full genome-wide screen to identify changes in the specific exon content of mRNA transcripts as well as overall gene expression. We identified a total of 287 genes that were up-regulated by at least 2-fold in response to androgen treatment and 263 genes that were down-regulated by at least 2-fold (Figure S1 and Table S1). GO analyses for the up-regulated genes identified GO terms relating to sterol and lipid metabolism, which were significantly enriched in the up-regulated dataset (Figure S1). We were unable to identify any significantly enriched terms in the down-regulated dataset. Compared with previous analyses (for example, see reference [22]), the response to androgens in this current study is thus typical of AR-regulated gene expression (Figure S1).

**Figure 1:**
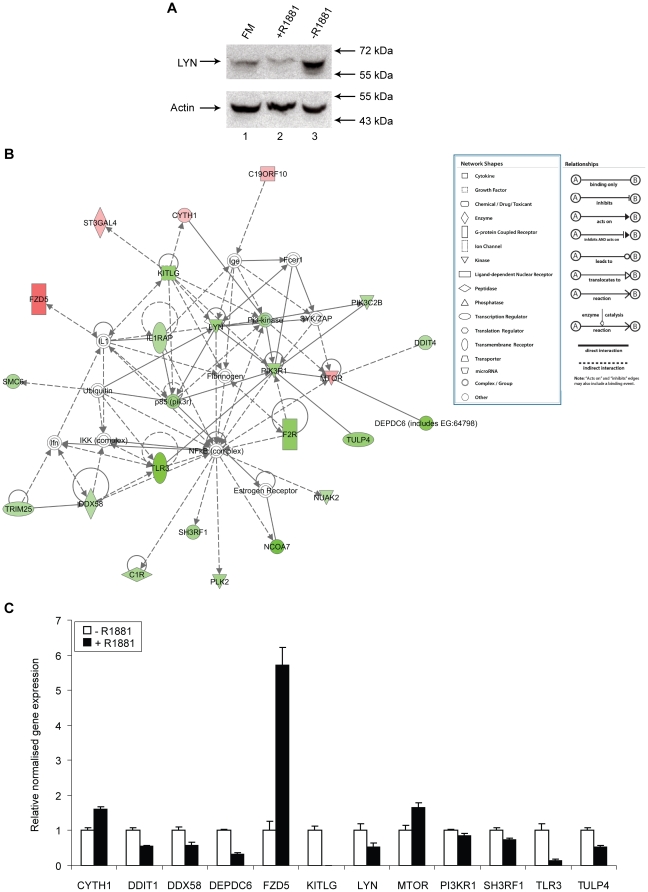
Expression of *LYN* and -associated genes are androgen-regulated in LNCaP cells. (A) Western analysis of LYN and actin (loading control) protein expression in cells grown in full medium, steroid-deplete medium, and following 24 hours of treatment with 10 nM R1881. (B) Ingenuity Pathway Analysis (IPA) of LYN-associated genes. Novel androgen-regulated genes identified in the present study are labelled in shades of red (up-regulated genes) or shades of green (down-regulated genes). (C) Expression of 12 novel androgen-regulated genes associated with *LYN* in steroid-deplete conditions (−, lanes 1–4) and after 24 hours of treatment with 10 nM R1881 (+, lanes 5–8) measured by multiplex RT-PCR. Levels of transcript expression were normalised to *GAPDH* levels, and data from experimental quadruplicates were used to obtain the mean ± SE for each transcript. A particularly high level of androgen-mediated gene activation was seen for the *FZD5* gene, which encodes a transmembrane Wnt5a receptor. Other confirmed up-regulated genes included *KITLG*, which encodes the Kit ligand upstream of *LYN* in the pathway, and *MTOR*, which encodes a key regulator of cell growth. Similarly, we confirmed down-regulation of the novel androgen-regulated genes *REDD1* (also known as *DDIT4*), which negatively regulates mTOR, and *DEPDC6* which encodes an mTOR interacting protein.

Approximately half of the androgen–responsive genes identified in our analysis have been previously found to be androgen-regulated (Table S1) in at least one of seven previously published datasets [16], [17], [18], [19], [20], [21], [22]. We did, however, additionally identify a subset of 226 novel genes transcriptionally regulated in response to androgens (Table S1) including *LYN*, which encodes a tyrosine kinase implicated in PCa cell biology [23]. Western analysis of lysates made from LNCaP cells grown in steroid-deplete medium and following 24 hours androgen treatment confirmed that the fall in *LYN* mRNA expression equated to a fall in LYN protein expression (Figure 1A). We next used the IPA “Core Analysis” function to identify clusters of novel androgen-regulated genes associated with, and including the tyrosine kinase LYN (Figure 1B). Using RT-PCR, we confirmed statistically-significant up-/down-regulation of *LYN* and eleven *LYN*-associated genes relative to *GAPDH* expression in each of the treatment samples as compared with controls (*p*<0.05) exactly as predicted by our microarray analysis (Figure 1B). The validation rate (100%) suggests that our microarray analysis is a robust description of androgen-dependent transcriptional changes in PCa cells.

**Figure 2:**
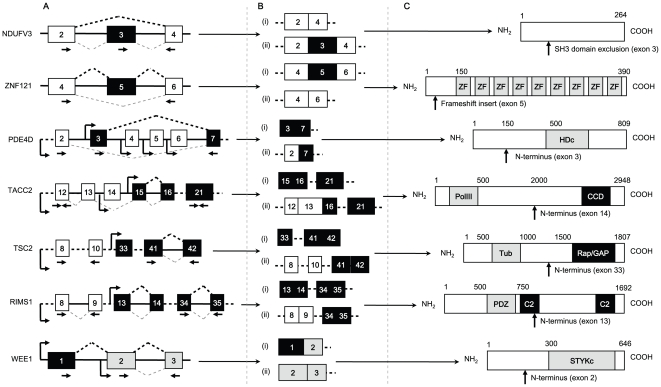
Overview of identified androgen-regulated mRNA isoforms. (A) Cartoon of intron/exon structure of gene, showing the splicing pattern and the positions of the primers (shown as arrows) used for RT-PCR. Genes are annotated with the androgen responsive splicing/promoter selection event above the gene (exons joined by broken lines in black), and constitutive event below the gene (exons joined by broken lines in grey). The androgen-regulated exons are shown in black, and weakly androgen-dependent exons indicated in grey. Constitutive exons are indicated in white. The positions of the primers used for RT-PCR validation are shown below exons as arrows, and predicted position of alternative promoters are shown as angled arrow. (B) For each gene a pair of alternative mRNA transcript isoforms is shown. (C) The predicted full-length proteins made from each of the genes are indicated, along with the position of conserved domains. These are abbreviated: ZF = zinc finger motif; HDc = metal dependent phosphohydroxylate with conserved HD motif; PolIII = DNA polymerase subunits gamma and tau; CC = coiled coil domain; Tub = tuberin superfamily; Rap/GAP = RAP/GTPase Activating Protein superfamily; PDZ = protein interaction and binding domain; C2 = protein kinase C conserved domain 2 Ca^2+^ binding motif; STYKc = STYK protein kinase domain.

**Figure 3:**
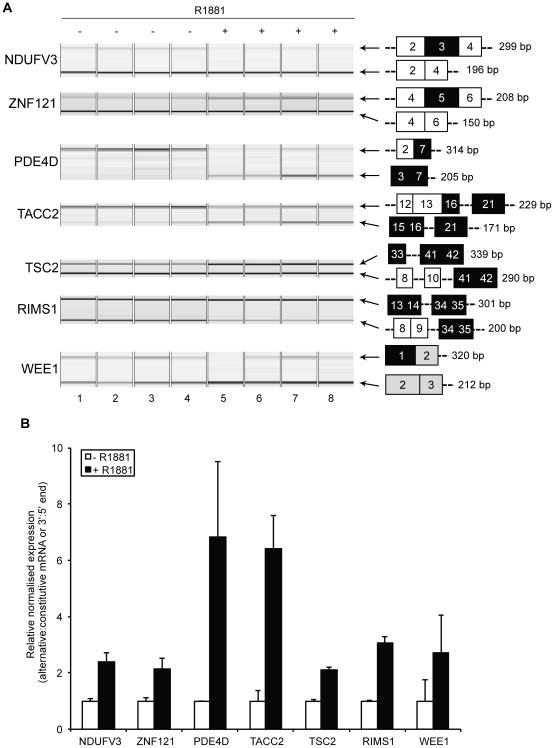
Androgens regulate expression of a number of alternative mRNA isoforms in LNCaP cells. (A) Capillary agarose gel electrophoretic analysis of RT-PCR products amplified from mRNA from cells grown either in steroid-deplete medium (lanes 1–4) or after 24 hours of treatment with 10 nM R1881 (lanes 5–8). The predicted mRNA isoforms detected are shown to the right of each gel exactly as summarised in Figure 2B. (B) Fluorescent quantification was performed to obtain the relative normalised expression ratio, which is the contribution of the PCR product representing the alternative (androgen-regulated) transcript/constitutive transcript or 3′ transcript end/5′ transcript end. Data from the experimental quadruplicates (gel image) were used to obtain the mean ± SE.

To detect changes in mRNA isoform ratios in response to androgen stimulation we applied PLIER/Iter-PLIER statistical analysis to exon level data along with several filtering criteria (see Materials and Methods). We identified a total of 1279 differentially expressed probesets (*p*<0.001), of which 325 (∼25%) mapped to well known alternative splicing events (n = 108; 8.4%), alternative first exons/5′ ends (n = 144; 11.3%) and alternative last exons/3′ ends (n = 73; 5.7%). The remaining probesets did not have obvious annotations. In view of previously-published data [14], [15], we focussed solely on cassette exons and changes in extreme 5′-ends of transcripts (alternative transcriptional initiation sites due to alternative promoter selection) for which there were full length annotated UCSC (University of California, Santa Cruz) gene sequences [24] available based on RefSeq or GenBank entries with known exon connectivity. Probe intensities of candidate transcripts were visualised at single exon level using EASANA to select candidates that might encode potentially androgen-regulated alternative mRNA isoforms for experimental verification. We identified a total of 30 candidate mRNA isoforms, of which 7 were positively validated in the array sample set as well as an independent dataset (Figures 2 and 3, and fully described below). In each validated case we found statistically significant differences (*p*<0.05) in actual biological ratios of constitutive and alternative (androgen-regulated) mRNA isoforms as monitored by multiplex RT-PCR and fluorescent quantification after capillary agarose gel electrophoresis (Figure 3). In each case, RT-PCR products were sequenced to verify the content of individual mRNA species.

### Identification of mRNAs containing androgen-regulated cassette exons

There are two fully annotated mRNA isoforms from the *NDUFV3* gene (RefSeq accession numbers NM_021075 and NM_001001503), which encodes a subunit of the NADH-ubiquinone oxidoreductase complex. Our analysis suggested differential androgen-regulated production of these two isoforms, with androgen-dependent skipping of a cassette exon (exon 3) within the mRNA transcript. Although no direct function for this gene product has been described in PCa, mitochondrial complex I, of which the NDUFV3 protein is a component, plays an important role in interferon-beta and retinoic acid-induced cancer cell death. The long (1095 nt; nucleotide) cassette exon within the *NDUFV3* transcript inserts coding information for a 365 amino acid (aa) peptide into the NDUFV3 protein sequence compared with the shorter mRNA isoform (Figure 2, NCBI NP_066553 and NP_001001503.1 respectively). Multiplex RT-PCR demonstrated expression of exon 3-included and exon 3-skipped transcripts in steroid-deplete conditions, and confirmed an androgen-dependent switch in exon 3 inclusion (Figure 3A, compare lanes 1–4 with 5–8). Following androgen treatment there was a ∼2 fold increase in the exon-skipped product relative to the exon-included product (Figure 3B).

Androgen-regulated inclusion of a cassette exon was also identified in the transcript derived from the gene encoding the putative transcription factor *ZNF121*. *ZNF121* encodes a 390 amino acid protein with 9 C_2_H_2_-type zinc finger motifs (Figure 2C), and is expressed as three alternative mRNA isoforms (UCSC mRNA isoforms uc010xkp.1, uc010dwt.2 and uc010xkq.1, respectively). Exon 2 is alternatively-spliced within the 5′ untranslated region (UTR), while inclusion of exon 5 interrupts the reading frame initiated in exon 4, and results in a frameshifted mRNA (Figure 2A and 3B). Using multiplex RT-PCR, exon 2 was found to be constitutively skipped in LNCaP cells (data not shown), but inclusion of the 58 nt exon 5 was significantly up-regulated in response to androgen treatment (Figure 3A, compare lanes 1–4 with lanes 5–8). In terms of functional outputs, cassette exon 5 introduces a frameshift to the ZNF121 protein sequence very close to the amine (N)-terminus of the encoded protein, and so may either destabilise the mRNA through the NMD pathway [25] or lead to production of a protein lacking zinc finger motifs (Figure 2C).

### Identification of mRNAs with distinct androgen-regulated 5′ ends

We also identified variant mRNA isoforms with alternative 5′ exons generated in response to androgen treatment from alternative transcriptional initiation sites due to alternative promoter selection. These included mRNA isoforms from the *PDE4D* gene, which encodes a cAMP-specific phosphodiesterase involved in signalling pathways, and has been recently identified as a proliferation-promoting factor up-regulated in clinical PCa [26]. The *PDE4D* gene is transcribed using an array of five alternative transcriptional initiation sites, including distinct promoter elements upstream of exon 1 (which is spliced onto exon 2 and then 7 (RefSeq NM_001165899) and upstream of exon 3 which is also directly spliced onto exon 7 (RefSeq NM_006203)) (Figure 3A) [26]. Using multiplex RT-PCR, LNCaP cells were found to initiate *PDE4D* transcription upstream of exon 3 in steroid-deplete conditions, and following androgen treatment a dramatic switch in transcriptional initiation was made to the promoter upstream of exon 1 (Figures 2 and 3). Both these *PDE4D* mRNA isoforms contain predicted ORFs for the conserved HDc motif of metal dependent phosphohydrolases (Figure 2C), but have different N-terminal sequences (NCBI NP_001159371.1 and NP_006194.2).

A switch between androgen-regulated mRNA isoforms with distinct 5′ ends was also identified for the *TACC2* gene, which encodes a centrosomal protein. Multiple different *TACC2* mRNA isoforms are transcribed from the *TACC2* gene, under the control of alternative internal promoters upstream of exons 14, 15, and 18 (Figure 2A) to give different mRNA isoforms with alternative exon contents [27]. Using multiplex RT-PCR using primers complementary to exons 12 (5′ end of the gene) and 21 (3′ end of the gene), we confirmed significantly increased levels of 3′ end of the *TACC2* gene as compared with the 5′ end following androgen treatment, suggesting androgen-regulated mRNA isoform production through an alternative internal AR-activated promoter (Figure 3). The use of a promoter upstream of exon 15 produces a variant mRNA isoform called *AZU-1*, in which the coding information for the coiled-coil domain is preserved (GenBank ID: AF176646.1). Although *TACC2* is a non-essential gene and its deletion does not cause cancer in mice, [28], [29], up-regulation of *AZU-1* has been implicated in advanced breast cancer [28]. We analysed expression of mRNA isoforms containing TACC2 exons 14, 15 and 18 which are the first exons of each of these annotated transcription units, and found that the promoter upstream of exon 15 is androgen-responsive, indicating *AZU-1* is an androgen-regulated transcription unit (data not shown).

Androgen-regulated selection of alternative 5′ ends were also detected in transcripts derived from the *TSC2*, *RIMS1* (*regulating synaptic membrane endocytosis 1*) and *WEE1* genes. *TSC2* encodes an important tumour suppressor protein tuberin, which regulates cell growth [30], [31]. The *TSC2* gene is complex and encodes a number of different mRNA isoforms with alternative exons, and our analysis predicted increased levels of transcription of the downstream region of the gene. Confirming this by multiplex RT-PCR using primers within exons 8 and 10 (5′ end) and 41 and 42 (3′ end), we observed an ∼2 fold increase in expression of 3′ end of the *TSC2* gene as compared with the 5′ end following androgen treatment (Figure 3A, compare lanes 1–4 with lanes 5–8). Known annotated mRNA isoforms on UCSC [24], [32] indicated two known alternative internal promoters upstream of *TSC2* exon 33 (Figure 2A), which might to respond to androgen stimulation (DP1 and DP2). The alternative *TSC2* transcript produced in response to androgen treatment contains coding information for the Rap/GAP domain of the full-length protein, but is missing the tuberin family domain (Figure 2C) (GenBank BAG52831.1).

Androgen-regulated mRNA isoforms were also predicted within the *RIMS1* gene, which is a member of the RAS gene superfamily, and encodes a protein that acts as a scaffold to regulate vesicle exocytosis. UCSC gene annotations [24], [32] indicate that *RIMS1* encodes a family of 11 different mRNA isoforms, transcribed downstream of 5 promoters. By multiplex RT-PCR using primers within exons 8 and 9 (5′ end) and exon 34 and 35 (3′ end), we observed an ∼3 fold increase in expression of 3′ end of the *RIMS1* gene as compared with the 5′ end following androgen treatment (Figure 3A compare lanes 1–4 with 5–8, and Figure 3B), most likely generated from transcriptional initiation from the UCSC annotated promoters upstream of exon 13 (Figure 2A) [24], [32]. The alternative transcripts produced in response to androgen treatment contain a predicted ORF, which excludes a PDZ domain involved in protein interactions and binding, and one calcium-binding (C2, protein kinase C conserved domain 2) domain (Figure 2C) compared with the full length protein.

In the above examples, androgen treatment led to alternative promoter selection and an increase in expression of an alternative mRNAs from a particular transcription start site. In contrast, we observed more subtle selective androgen-dependent alternative promoter usage within the androgen down-regulated *WEE1* gene. *WEE1* encodes an important nuclear tyrosine kinase, which functions as a negative regulator of cell cycle progression and inhibits Cdc2-Cyclin B activity and entry into mitosis. UCSC annotations and published analyses of mRNA isoforms [24], [32], [33] indicate that *WEE1* encodes two alternative mRNAs initiating upstream of exon 1 (isoform 1 encoding full length Wee1 protein) or exon 2 (isoform 2 encoding a shorter protein Wee1i with an alternative N-terminus) (Figure 2). Both the long and the shorter forms of WEE1 protein contain the STY protein kinase domain (STYKc), but the shorter WEE1i does not contain the 214 aa N-terminus domain which mediates ubiquitin-dependent degradation of WEE1 protein to trigger mitotic entry [33]. Multiplex RT-PCR indicated that both *WEE1*-derived transcripts were down-regulated following androgen treatment, however the longer mRNA isoform (RefSeq NM_003390) was down-regulated to a greater extent as compared with the shorter mRNA isoform (RefSeq NM_001143976) (Figure 3A, compare lanes 1–4 with 5–8). The relative (∼3 fold) increase in abundance of the shorter mRNA isoform (and possibly Wee1i protein) following androgen treatment may provide the cell with constitutive WEE1-like kinase activity (protein NCBI NP_001137448.1) to regulate androgen-induced cell cycle progression (Figure 3B).

### Novel mRNA isoforms are regulated by endogenous androgens and expressed in benign prostate epithelial cells as well as clinical PCa

We validated the above findings using a natural androgen (dihydrotestosterone; DHT) at physiological concentrations (1 nM and 10 nM), as well R1881 at 1 nM concentration, which is thought to positively affect cell proliferation [21] (Figure S2). RT-PCR was performed using total RNA extracted from LNCaP cells grown in steroid-deplete medium and following 24 hours of treatment with different concentrations of R1881 or DHT. The absolute magnitude of the actual biological ratios of constitutive and alternative (androgen-regulated) mRNA isoforms varied for each transcript depending on the dose of R1881 and DHT. Although expression of the alternative mRNA isoform encoded by *WEE1* was not affected by 1 nM R1881, differential expression was observed following treatment with 1 nM and 10 nM DHT. DHT (at both 1 nM and 10 nM concentrations) did not have an effect on expression of the alternative mRNA isoform derived from *RIMS1*, however expression changes were seen with 1 nM R1881. For other transcripts, an overall trend was observed towards relative increased expression of androgen-regulated mRNA isoforms in keeping with the data with 10 nM R1881. These findings suggest that the functional cellular response to androgens mediated by alternative mRNA isoform expression may be dependent on the local (physiological) concentration of endogenous ligand.

To determine whether the identified androgen-dependent mRNA isoforms are cancer-specific and cell line-specific, we examined the expression of alternative mRNAs in other tumourigenic androgen-dependent (CWR22Rv1) and androgen-independent (LNCaP-AI, LNCaP-cdxR) PCa cell lines, as well as a non-tumourigenic androgen-independent prostate epithelial cell line (BPH-1) (Figure S3). Since we were examining the steady-state levels of mRNA expression (rather than dynamic changes in response to androgens), we focussed on the alternative mRNAs (derived from *NDUFV3*, *ZNF121*, *PDE4D*, and *WEE1*) that could be specifically detected with our RT-PCR assays in steady-state conditions (culture medium containing androgens). Androgen-responsive exons were not exclusive to cancer cells (expressed in BPH-1 cells), nor to PCa cell lines that exhibit an androgenic growth response (LNCaP, and CWR22Rv1). In addition, alternative mRNA isoforms were present in LNCaP cells cultured in medium devoid of steroids (LNCaP-AI) and with anti-androgens (LNCaP-cdxR) (Figure S3A). To demonstrate that AR is required to mediate androgen-regulated expression of alternative mRNAs, we examined expression of alternative exons in PCa cell lines that do not exhibit an androgenic growth response (DU145, PC-3, PC-3M) (Figure S3B). Although alternative exons were expressed in these cell lines, treatment with androgens did not alter their expression levels.

Finally, we studied expression of alternative mRNA isoforms (derived from *NDUFV3*, *ZNF121*, *PDE4D*, and *WEE1*) in prostate biopsies from patients with PCa (Figure S3C), and found that although alternative exons were expressed in clinical PCa, there was no association with disease progression (*p*>0.05). Taken together, the above data suggest that in addition to androgens, expression of alternative mRNAs may be regulated by other *trans*-acting factors as well as *cis*-regulatory elements in a cell type- and tissue-specific manner.

### Expression of novel mRNA isoforms is both directly and indirectly regulated by androgens

**Figure 4:**
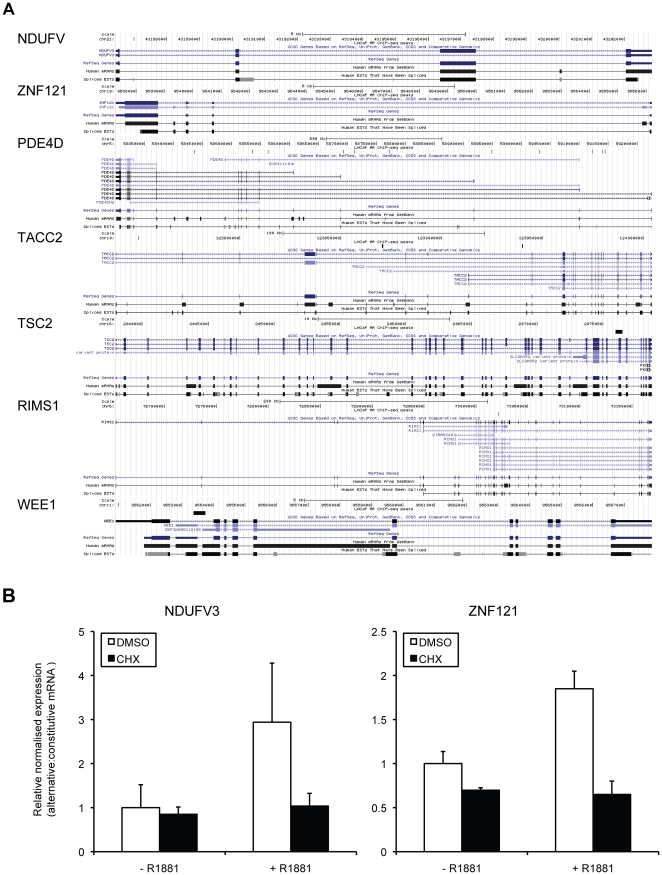
Expression of novel mRNA isoforms is both directly and indirectly regulated by androgens. (A) UCSC Genome Browser showing chromosomal locations and annotated transcript details for each novel androgen-regulated alternative mRNA isoform. Custom tracks (black bars below “LNCaP AR ChIP-seq peaks”) for each gene show locations of AR binding site(s) within the LNCaP genome as determined by ChIP-Seq [4]. (B) Fluorescent quantification of capillary agarose gel electrophoretic analysis of RT-PCR products amplified from mRNA from LNCaP cells pretreated for 1 hour with vehicle (DMSO) or 1 g/ml cycloheximide (CHX) prior to addition of 10 nM R1881 for 24 hours. The relative normalised expression ratio is the contribution of the PCR product representing the alternative (androgen-regulated) transcript/constitutive transcript. Data from four independent replicates were used to obtain the means ± SE.

Recent studies have used chromatin immunoprecipitation (ChIP) and direct next generation sequencing (ChIP-Seq) for unbiased mapping of AR genomic binding sites in LNCaP cells in response to androgens [3], [34]. To determine whether the association of AR within candidate genes identified in our study may drive the expression of alternative mRNA isoforms, we compared the previously published AR binding profiles [3], [34] to the predicted locations of alternative events (Figures 4A and S4). The genomic loci of AR binding sites were uploaded to UCSC genome browser [24], and interrogated for positively-validating genes of interest from our present study (Figure 2). In keeping with our hypothesis, AR binding (Figure 4A, LNCaP AR ChIP-seq peaks, and Figure S4) was observed close to alternative promoters predicted to yield variant androgen-regulated mRNA isoforms with alternative 5′ exons generated from alternative transcriptional initiation sites (*PDE4D*, *TACC2*, *TSC2*, *RIMS1*, and *WEE1*). However, we did not observe AR binding within genes encoding transcripts identified to possess androgen-regulated cassette exons (*NDUFV3* and *ZNF121*) suggesting that these splicing events may be due to the indirect effect of androgens.

To test this hypothesis, we co-treated LNCaP cells with or without 10 nM R1881 (as above) in conjunction with the translational inhibitor cycloheximide or vehicle (dimethylsulfoxide; DMSO) (Figure 4B). Using multiplex RT-PCR, we monitored exon skipping and exon inclusion within transcripts derived from *NDUFV3* and *ZNF121*, respectively. Cycloheximide completely inhibited androgen-dependent *NDUFV3* exon 3 skipping and *ZNF121* exon 5 inclusion. These data indicate that the effect of androgens on *NDUFV3* and *ZNF121* splicing is indirect and requires intermediate protein synthesis.

### Ligand-activated AR is rapidly recruited to an alternative internal promoter within the *TSC2* gene

**Figure 5:**
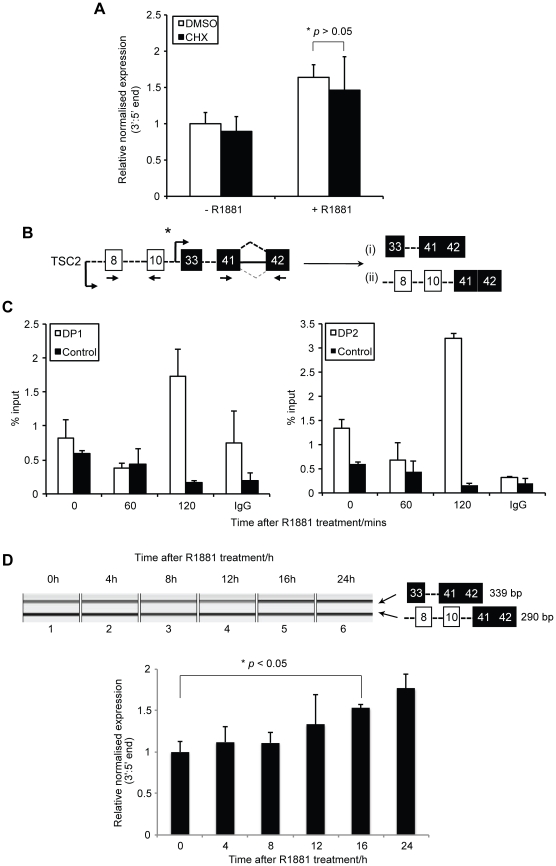
The association of AR with an alternative internal promoter region within *TSC2* correlates with dynamic expression of an alternative TSC2 mRNA. (A) Fluorescent quantification of capillary agarose gel electrophoretic analysis of RT-PCR products amplified from mRNA from LNCaP cells pretreated for 1 hour with vehicle (DMSO) or 1 g/ml cycloheximide (CHX) prior to addition of 10 nM R1881 for 24 hours. The relative normalised expression ratio is the contribution of the PCR product representing the 3′ transcript end/5′ transcript end. Data from four independent replicates were used to obtain the means ± SE. (B) Cartoon of intron/exon structure of *TSC2* gene, showing alternative promoter upstream of exon 33. The androgen-regulated exons are shown in black, and constitutive exons are indicated in white. The position of both alternative promoters DP1 and DP2 controlling expression of the shorter TSC2 variant mRNA is asterisked. (C) LNCaP cells cultured in steroid-deplete medium for 47 hours were treated with 10 nM R1881 for up to 120 min at 60 min intervals prior to ChIP analysis using anti-AR antisera. Input and immunoprecipitated material were analysed by real-time PCR, using primers for the DP1 (left panel) and DP2 (right panel) regions of the downstream promoter regions upstream of exon 33, as well as a non-AR-binding region within *GREB1* (control). Data shown are means ± SE from three independent experiments. (D) Capillary agarose gel electrophoretic analysis (upper panel) of RT-PCR products amplified from mRNA from cells grown either in steroid-deplete medium (lane 1) or following treatment with 10 nM R1881 (lanes 2–6) at timepoints upto 24 hours as shown. The predicted mRNA isoforms detected are shown to the right of each gel exactly as summarised in Figure 2B. Fluorescent quantification (lower panel) was performed to obtain the relative normalised expression ratio, which is the contribution of the PCR product representing the 3′ transcript end/5′ transcript end. The gel image (upper panel) is representative of three independent replicates from which data were used to obtain the means ± SE (lower panel).

To determine whether AR can directly regulate alternative mRNA expression from alternative transcription start sites, we explored the association of AR with the *TSC2* gene. To test our hypothesis that the alternative *TSC2* mRNA isoform is directly androgen-regulated, we firstly examined expression of TSC2 mRNAs from LNCaP cells co-treated with or without 10 nM R1881 (as described above) in conjuction with cycloheximide or vehicle (DMSO) (Figure 5A). Cycloheximide did not have any significant effect (*P*>0.05) on expression of androgen-induced mRNA isoform expression indicating that the ligand-dependent effect of AR on the *TSC2* gene is direct and does not require intermediate protein synthesis.

To confirm this hypothesis, we examined the dynamic recruitment of ligand-activated AR to alternative internal promoters (Figure 4A) of *TSC2* by ChIP at early timepoints following androgen stimulation. LNCaP cells were cultured in steroid-deplete medium to quiesce AR activity, and subsequently treated with 10 nM R1881 as the only available steroid and AR ligand. Chromatin was harvested at 60-minute intervals following androgen treatment, and subjected to immunoprecipitation using anti-AR antisera. Recovered genomic DNA was analysed by qPCR to examine recruitment of AR protein to the DP1 and 2 (Figure 4B, left and right panels, respectively) as well as a region of the *TSC2* gene that was not predicted to bind AR. Maximal recruitment of AR to both DP1 and 2 was observed after 120 minutes following androgen stimulation, but not to the non-AR-binding region within *GREB1*. Overall these data show that AR is dynamically recruited to DP1 and 2 within *TSC2*, in response to ligand activation. Due to close proximity of the promoters (separated by 524 nucleotides), it was difficult to cleanly resolve the association of AR-promoter association.

Since AR-dependent gene expression is a dynamic process [17], we measured the expression of the shorter AR-regulated TSC2 isoform by RT-PCR at timepoints up to 24 hours following androgen stimulation (Figure 4C). We observed an increasing temporal trend in expression of the alternative *TSC2* isoform, which reached statistical significance at 16 hours following androgen stimulation (*p*<0.05). Taken together, these data suggest that recruitment of ligand-activated AR to internal androgen-regulated promoters controls expression of an alternative *TSC2* mRNA isoform. Although we observed a lag in alternative isoform expression ratios following AR recruitment to the DP1 and 2 promoters, this is likely explained by the stability of the constitutive mRNA isoform and the time taken to establishment of steady-state levels.

## Discussion

In the present study, we have undertaken gene expression analysis at exon level resolution to identify androgen-regulated production of both novel genes and endogenous variant mRNA isoforms. Gene expression pathway analysis revealed clusters of novel androgen-regulated genes associated with, and including the tyrosine kinase LYN, which is required for PCa cell proliferation [23]. Our experimental data indicates that *LYN* expression is normally repressed by androgens both at the mRNA and the protein level. We also identified androgen-dependent up-regulation of the LYN-associated kinase mTOR. mTOR kinase activity has been previously found to be up-regulated in PCa cells in response to androgens [35], and the mTOR pathway is important in clinical PCa, particularly in tumours lacking expression of *PTEN* (phosphatase and tensin homolog). Since AR gene targets can remain active in CRPCa, the mTOR complex may be a possible therapeutic target for CRPCa [35], [36].

Exon resolution analyses further identified a number of mRNA isoform switches that occur in response to androgen treatment, including selection of alternative 5′ exons and inclusion of cassette exons. The oestrogen receptor (ER) has been recently shown to regulate expression of many mRNA isoforms from alternative transcriptional initiation sites as are result of alternative promoter selection [13]. ER was found to associate with alternative promoters regions, and ER-regulated alternative mRNA isoforms influenced breast cancer cell behaviour. Similarly, based upon published data [3], we predicted the association of AR to alternative promoter regions within candidate genes, and confirmed AR binding to an alternative promoter region (comprising DP1 and 2) within the *TSC2* gene. This association directly regulated the dynamic accumulation of a shorter *TSC2* mRNA predicted to encode a 508 aa C-terminus peptide up to 24 hours after androgen addition (GenBank protein accession: BAG52831.1). This peptide lacks an interaction domain needed to form a heterodimer with TSC1 (hamartin), which is required to regulate mTOR kinase function. Although primarily characterised as an upstream regulator of mTOR, TSC2 also has been associated with mitotic structures and cell cycle control, and in particular the C-terminus of the full-length protein has been localised to the nucleus and associated with the transcriptional activity of FOXO transcription factors [37]. We are currently exploring our hypothesis that this novel AR-regulated TSC2 mRNA isoform might play an important role in mTOR regulation in PCa cells.

In principle, ExonArrays should provide an extremely accurate measure of gene expression [38] since data are gathered along the full length of the transcript. Compared with other published datasets on androgen-regulated gene expression [16], [17], [18], [19], [20], [21], [22], the results of our analysis have the most extensive overlap with other microarray analyses. Although ∼50% of gene expression changes in our dataset were novel, these findings may be in part due to experimental differences between previously published studies rather than platform-specific differences (Table S2). Indeed, a more detailed analysis suggested that many of the novel hits are in fact functionally related to known androgen-regulated genes (see Table S2, column 5). Of the 1279 putative androgen-regulated alternative events identified, only ∼25% mapped to known alternative splicing events or alternative first/last exons. Furthermore, the low positive validation rate (∼23%) highlights the technical challenges of using ExonArrays to identify alterations in alternative mRNA isoform expression on a background of large changes in gene expression [15]. Taken together, our analyses suggest that the bulk of androgen-regulated effects on the transcriptome are at the level of overall gene expression rather than alterations in the expression of variant mRNAs.

In summary, we have found that androgens not only have quantitative effects on gene expression but also have some qualitative effects on transcript composition. While different molecular mechanisms may underlie androgen-regulated expression of alternative mRNAs (including alternative splicing, transcriptional initiation or RNA stability) in a cell line- and tissue-specific manner, our data suggest that androgens directly regulate expression of alternative 5′ exons (as a result of promoter selection), and indirectly regulate alternative splicing. The androgen-dependent variant transcripts identified in this study are predicted to yield protein isoforms that may mediate the cellular response to androgens, although the functional roles of these mRNA isoforms in clinical PCa is as yet unknown, and warrants further investigation.

## Materials and Methods

### Antibodies

The following antibodies were used: anti-LYN mouse monoclonal antibody (65038-1-1 g, Proteintech), anti-AR rabbit antisera (C19, Santa Cruz Biotechnology), anti-actin rabbit antisera (A-2066, Sigma), and normal rabbit IgG (sc-2027, Santa Cruz Biotechnology).

### Cell line samples for array analysis and RT-PCR validation

All cells were grown at 37°C in 5% CO_2_. LNCaP cells (CRL-1740, ATCC) were maintained in RPMI-1640 with L-Glutamine (PAA Laboratories, R15-802) supplemented with 10% Fetal Bovine Serum (FBS) (PAA Laboratories, A15-101). For details of other cell lines see Materials and Methods S1. Where indicated, medium was supplemented with 10% dextran charcoal stripped FBS (PAA Laboratories, A15-119) to produce a steroid-deplete medium. In experiments performed in quadruplicate, LNCaP cells were cultured for 72 hours, following which 10 nM synthetic androgen analogue methyltrienolone (R1881) (Perkin–Elmer, NLP005005MG) was added (treatment; n = 4) or absent (control; n = 4) for 24 hours. Cells were then harvested and total RNA extracted using TRIzol (Invitrogen, 15596-026) according to manufacturer's instructions as previously described [9]. To ensure array findings were not due to batch effects and to confirm the reproducibility of validation methodologies, the above experiment was performed independently in triplicate and all samples analysed by qPCR and RT-PCR in an identical manner. Where indicated, LNCaP cells were pre-treated for 1 hour with vehicle (dimethylsulfoxide; DMSO) (Sigma, C1988) or 1 µg/ml cycloheximide (Sigma, D2438) prior to addition of 10 nM R1881 for 24 hours as previously described [39].

### Clinical samples

For details of clinical samples see Materials and Methods S1.

### RNA purification and array hybridization

Total RNA from treatment and control experiments (n = 8) was purified using RNeasy Plus Kit (Qiagen, 74134), which includes genomic DNA (gDNA) eliminator column to remove contaminating DNA accordingly to the manufacturer's instructions. Ribosomal RNA (rRNA) reduction was performed using the RiboMinus Kit (Invitrogen, K1550-02). RNA quality was verified by analysis on the 2100 Bioanalyzer (Agilent). One microgram of total RNA was labelled according to the GeneChip Whole Transcript (WT) Sense Target Labelling Assay provided by the manufacturer (Affymetrix, 900670) and hybridized to Human Exon 1.0 ST Arrays (Affymetrix, 900649) overnight before scanning in an Affymetrix GCS 3000 7G scanner. Raw data has been deposited at Gene Expression Omnibus (http://www.ncbi.nlm.nih.gov/geo/) under accession number GSE 32875 and all details are MIAME compliant.

### Data analysis

Following Quality Assessment of datasets [40], gene expression values were obtained by quantile-normalisation using Robust Multichip Analysis (RMA) with core probesets (228,940 probesets), and antigenomic background probes [40]. After summarization, the Student's T-test was used to detect differential expression between control and treatment arrays. Only probe sets that were detected above background (defined by detection above background; DABG *p*≤0.01) were included in the analysis. The False discovery rate (FDR) was calculated to make corrections of multiple testing, using the Q-value program [41], with *q*<0.005 taken to indicate statistical significance. Gene Ontology (GO) annotations for all human genes obtained from http://www.geneontology.org, was compared with annotations for the set of genes up-regulated following androgen treatment. We calculated whether a term occurs proportionally more in the input set than in the entire genome and determined if the difference was significant using a Fisher's Exact test which returned a *P*-value. FDR *q* = 0.05 was used to determine a cut off, and the obtained list of terms was filtered so that terms that were significant only because one (and only one) of their descendant terms was significant were removed. Gene lists were uploaded to the web-based Ingenuity Pathway Analysis (IPA; Ingenuity Systems) software programme, and the “Core Analysis” function was used to study direct and indirect regulatory relationships between genes and their known biological functions.

For detection of alternative splicing, the student's T-test was performed on “normalized” probeset intensities, which were obtained by removal of core meta probeset intensities (gene level estimates) from extended probeset intensities (exon level estimates). Iter-PLIER algorithm was used for gene level estimates, and the PLIER algorithm was used for exon level estimates [40]. To improve the accuracy of gene expression estimation, only those meta probesets with ≥11 core probes were used. Other genes were excluded from the analysis. 16,309 of 17,881 meta probesets passed this filtering. After the T-test for differential splicing, the following filtering criteria were applied to identify differentially spliced probe sets: a) The gene is expressed in both conditions and DABG *p*≤0.01 at least in three replicates in each condition; b) The reliability of gene level estimates was tested by extracting the probe intensities for each meta probeset, obtaining an average across replicates for each condition, and ensuring good correlation (Pearson product-moment and Spearman's rank correlation coefficient≥0.7) between conditions; c) Gene expression between conditions should not exceed 10 fold (3.32 in the log2 scale); d) The T-test of normalized probe set intensities yields *p*≤0.001 (or *p*≤0.0005); e) The exon must have detectable expression at least in one condition. More specifically, the DABG *P*-value at the probe set level must be ≤0.01 in three replicates in either condition. In addition, in this condition, the probe set intensity must be in an appropriate range compared with gene expression level (between 1/5 and 5, or −2.32 and 2.32 in the log2 scale).

Differentially spliced probe sets were mapped to alternative splicing events (including cassette exons, alternative 5′ and 3′ splice sites and mutually exclusive exons), as well as alternative first exons (alternative 5′ ends/promoter selection) and alternative last exons (alternative 3′ ends/polyadenylation). Alternative splicing events were obtained from the dbCASE database (http://rulai.cshl.edu/dbCASE) and alternative first and last exons were derived from UCSC known genes. We limited our analysis to alternative transcripts which had full length annotated UCSC/RefSeq sequences with known exon structures [42]. Probe data were visualised in their gene context using the EASANA visualisation module (GenoSplice Technology), and candidate transcripts were inspected using EASANA and the UCSC Genome Browser. We focused our search on cassette exons and changes in 5′-ends of transcripts selecting candidates for RT-PCR validation. For positively-validated targets, changes in peptide features of translated proteins resulting from alternative mRNA isoforms were identified using FAST-DB [27], [43], identifying conserved domains using the NCBI webpage (http://www.ncbi.nlm.nih.gov/sites/entrez) and the UCSC Genome Browser (http://genome.ucsc.edu/) [24].

### qPCR, RT-PCR and sequencing

cDNA was generated by reverse transcription of 1 µg of total RNA using the Superscript VILO cDNA synthesis kit (Invitrogen, 11754-050) according to manufacturers instructions. Quantitative PCR (qPCR) (Applied Biosystems 7900HT) was performed in triplicate on cDNA and gDNA templates using Platinum SYBR Green qPCR Super Mix-UDG with ROX (Invitrogen, 11744-500) as previously described [44]. PCR using 1 µl of 20-fold diluted cDNA was performed for 30 cycles with *Phusion* High Fidelity DNA Polymerase (Finnzymes, F-530S). All primer sequences are listed in Table S3. RT-PCR products were initially analyzed on 2–3% agarose gels, and subsequently using the QIAxcel capillary gel electrophoresis system (Qiagen), which was also used for image acquisition. Ratios between products representing transcripts of interest multiplexed with *glyceraldehyde-3-phosphate dehydrogenase* (*GAPDH*) controls as well as mRNA isoforms were determined using QIAxcel software. The relative normalised gene expression was calculated as a ratio of the fluorescent intensity of the band representing amplification of the transcript of interest/fluorescent intensity of band representing amplification of *GAPDH*). The relative normalised expression ratio was calculated as follows: fluorescent intensity of band representing the alternative (androgen-regulated) transcript/fluorescent intensity of band or fluorescent intensity of band representing 3′ transcript end/5′ transcript end. The independent sample T-Test was employed to identify statistically significant differences in total transcript and alternative mRNA isoform expression with *p*<0.05 taken to indicate statistical significance. To confirm the identities of RT-PCR products distinct gel bands were purified with the QIAquick Gel Extraction kit (Qiagen, 28704), cloned using a CloneJET PCR Cloning Kit (Fermentas Life Sciences, K1231) and positive transformants were sequenced (GATC Biotech) using a T7 sequencing primer.

### Chromatin Immunoprecipitation (ChIP) assays

ChIP assays were performed as previously described [44], using 2 µg anti-AR antisera and normal IgG controls. qPCR (Applied Biosystems 7900HT) was performed on inputs and recovered material to assess AR association to DP (downstream promoter) 1 and 2, and non-AR-binding region within *GREB1* (chromosome positions chr16:2132598–2132747, chr16:2133122–2133271, and chr2:11597650–11597669, respectively). All experimental results shown are the means of at least three independent experiments ± standard error (SE).

## Supporting Information

Materials and Methods S1 (DOCX)

Figure S1 **Analysis of androgen-regulated gene expression.** (A) Experimental validation of changes in *KLK3* gene expression. LNCaP cells were grown in steroid-deplete medium for 72 hours followed by treatment (n = 4) or absent (n = 4) for 24 hours with 10 nM R1881. *KLK3* and *GAPDH* transcript expression was analysed by qPCR analysis *KLK3* transcript levels were normalised to *GAPDH* levels. Data from qPCR triplicates were used to obtain the mean ± SE for each sample. (B) Principal Component Analysis (PCA) mapping of microarray datasets show segregation into control (Ctr) and androgen treatment (And) conditions. (C) Scatterplot showing gene expression in steroid-deplete medium and following androgen treatment. Differentially-expressed genes are shown in red (≥2-fold change in gene expression with a *q*-value≤0.005). (D) Gene Ontology (GO) analysis of genes up-regulated in response to androgen treatment.
(EPS)

Figure S2 **Dose-independent androgen-regulation of alternative mRNA isoforms occurs with synthetic and natural androgens.** Fluorescent quantification of capillary agarose gel electrophoretic analysis of RT-PCR products amplified from mRNA from LNCaP cells grown in steroid-deplete medium or after 24 hours of treatment with different concentrations of synthetic (R1881) and natural (dihydrotestosterone; DHT) androgens. The relative normalised expression ratio is the contribution of the PCR product representing the alternative (androgen-regulated) transcript/constitutive transcript or 3′ transcript end/5′ transcript end. Data from three independent replicates were used to obtain the means ± SE.
(TIF)

Figure S3 **Androgen-regulated mRNAs isoforms are expressed in benign and malignant prostate cell lines and cases of clinical PCa.** (A) Capillary agarose gel electrophoretic analysis of RT-PCR products amplified from mRNA from prostate epithelial cell lines. LNCaP-AI and LNCaP-cdxR cells are able to grow in steroid-deplete medium and medium containing anti-androgens (bicalutamide), respectively. The predicted mRNA isoforms detected are shown to the right of the gel exactly as summarised in Figure 2B. (B) Fluorescent quantification of capillary agarose gel electrophoretic analysis of RT-PCR products amplified from mRNA from PCa cell lines that do not exhibit an androgen-dependent transcriptional response (PC-3, PC-3M, DU145). Cells were grown either in steroid-deplete medium or after 24 hours of treatment with 10 nM R1881. Data from three independent replicates were used to obtain the means ± SE. (C) Agarose gel electrophoretic analysis of RT-PCR products amplified from mRNA from clinical prostate samples from patients with hormone-naïve (HN) (odd lanes) and following development of castration-resistant (CR) (even lanes) disease (n = 10). The predicted mRNA isoforms detected are shown to the right of the gel exactly as summarised in Figure 2B. As a control to demonstrate mRNA integrity and overall levels of gene expression between patients, equal levels of *GAPDH* could be detected in each of the patient mRNA samples. Densitometric band quantification was performed to obtain the relative normalised expression ratio, which is the contribution of the PCR product representing the alternative (androgen-regulated) transcript/constitutive transcript (data not shown). M = 1 Kb plus DNA ladder (Invitrogen).
(TIF)

Figure S4 **Expression of novel mRNA isoforms is both directly and indirectly regulated by androgens.** (A) UCSC Genome Browser showing chromosomal locations and annotated transcript details for each novel androgen-regulated alternative mRNA isoform. Custom tracks (black bars below “Human_LNCaP_AR_R1881(16h)_Chinnaiyan”) for each gene show locations of AR binding site(s) within the LNCaP genome as determined by ChIP-Seq [34].
(TIF)

Table S1 **Androgen-regulated gene expression changes.** Individual gene expression changes following androgen treatment are shown with fold change predicted based upon microarray prediction, and the actual fold change where measured by RT-PCR. Androgen-regulated genes identified previously published studies are indicated. The functions and associated processes of each gene are annotated where known.
(XLS)

Table S2 **Overlap of identified androgen-regulated genes with previously-published datasets.**
(DOC)

Table S3 **Genomic sequence of oligonucleotide primers.** (A) qPCR for gene expression analysis (B) RT-PCR for splicing analysis (C) RT-PCR for gene expression analysis (D) qPCR for ChIP analysis. Where possible, primers were designed so that amplicons spanned exon junctions. Where shown predicted amplicon sizes was obtained using the *in silico* PCR program http://genome.ucsc.edu/index.html?org=Human&db=hg18&hgsid=142437216.
(DOC)
