# Supplementary material for: Identification of Novel Androgen-Regulated Pathways and mRNA Isoforms through Genome-Wide Exon-Specific Profiling of the LNCaP Transcriptome
Source: PLoS One. 2011 Dec 14;6(12):e29088. doi: 10.1371/journal.pone.0029088 (PMC3237596; doi:10.1371/journal.pone.0029088)
Supplement: Materials and Methods S1 — (DOCX) [file pone.0029088.s001.docx]

**Supplementary Materials and Methods S1**

**Cell lines:** PC-3 (CRL-1435, ATCC), PC-3M [45], CWR22Rv1 (CRL-2505, ATCC), DU145 (HTB-81, ATCC), and BPH-1 cells [46] were maintained in RPMI-1640 with L-Glutamine supplemented with 10% FBS. LNCaP-AI and LNCaP-cdxR were derived from LNCaP parental cells and maintained as previously described [47,48].

**Clinical Samples**: Full ethical approval was obtained for all human sample collections from the Northumberland, Tyne and Wear NHS Strategic Health Authority Local Research Ethics Committee (Ref: 2003/11). Clinical samples were obtained as previously described [49]. The clinical cohort used consisted of 10 matched hormone-naïve (HNPCa) and relapsed castrate-resistant PCa (CRPCa) sample pairs. PCR using 1 µl of 20-fold diluted cDNA was performed for 38 cycles with Phusion High Fidelity DNA Polymerase. RT-PCR products were analyzed both on 2–3 % agarose gels and images were acquired using GeneSnap v7.05(b) (Syngene). Ratios between products representing mRNA isoforms of interest were determined by densitometry using using GeneTools v.4.00(a) (Syngene) image analysis software. The relative normalised expression ratio was calculated as follows: fluorescent intensity of band representing the alternative (androgen-regulated) transcript/fluorescent intensity of band or fluorescent intensity of band representing 3’ transcript end/5’ transcript end.

**References**

45. Kozlowski JM, Fidler IJ, Campbell D, Xu ZL, Kaighn ME, et al. (1984) Metastatic behavior of human tumor cell lines grown in the nude mouse. Cancer Res 44: 3522-3529.

46. Hayward SW, Dahiya R, Cunha GR, Bartek J, Deshpande N, et al. (1995) Establishment and characterization of an immortalized but non-transformed human prostate epithelial cell line: BPH-1. In Vitro Cell Dev Biol Anim 31: 14-24.

47. Halkidou K, Gnanapragasam VJ, Mehta PB, Logan IR, Brady ME, et al. (2003) Expression of Tip60, an androgen receptor coactivator, and its role in prostate cancer development. Oncogene 22: 2466-2477.

48. Rigas AC, Robson CN, Curtin NJ (2007) Therapeutic potential of CDK inhibitor NU2058 in androgen-independent prostate cancer. Oncogene 26: 7611-7619.

49. Armstrong K, Ahmad I, Kalna G, Tan SS, Edwards J, et al. (2011) Upregulated FGFR1 expression is associated with the transition of hormone-naive to castrate-resistant prostate cancer. Br J Cancer 105: 1362-1369
