# Supplementary figures and images for: Identification of Novel Androgen-Regulated Pathways and mRNA Isoforms through Genome-Wide Exon-Specific Profiling of the LNCaP Transcriptome
Source: PLoS One. 2011 Dec 14;6(12):e29088. doi: 10.1371/journal.pone.0029088 (PMC3237596; doi:10.1371/journal.pone.0029088)

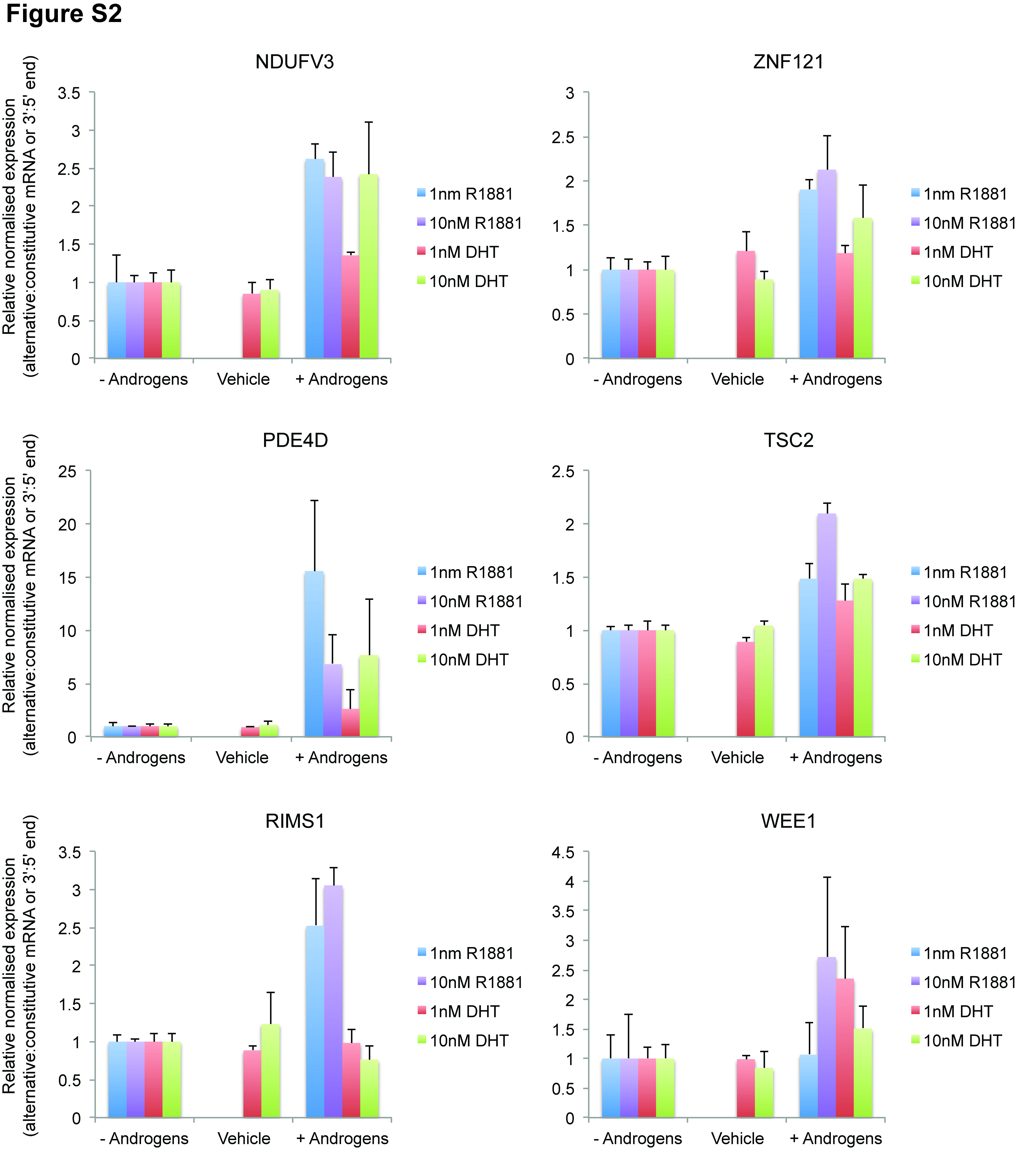

Supplement: Figure S2 — Dose-independent androgen-regulation of alternative mRNA isoforms occurs with synthetic and natural androgens. Fluorescent quantification of capillary agarose gel electrophoretic analysis of RT-PCR products amplified from mRNA from LNCaP cells grown in steroid-deplete medium or after 24 hours of treatment with different concentrations of synthetic (R1881) and natural (dihydrotestosterone; DHT) androgens. The relative normalised expression ratio is the contribution of the PCR product representing the alternative (androgen-regulated) transcript/constitutive transcript or 3′ transcript end/5′ transcript end. Data from three independent replicates were used to obtain the means ± SE. (TIF) [file pone.0029088.s003.tif]

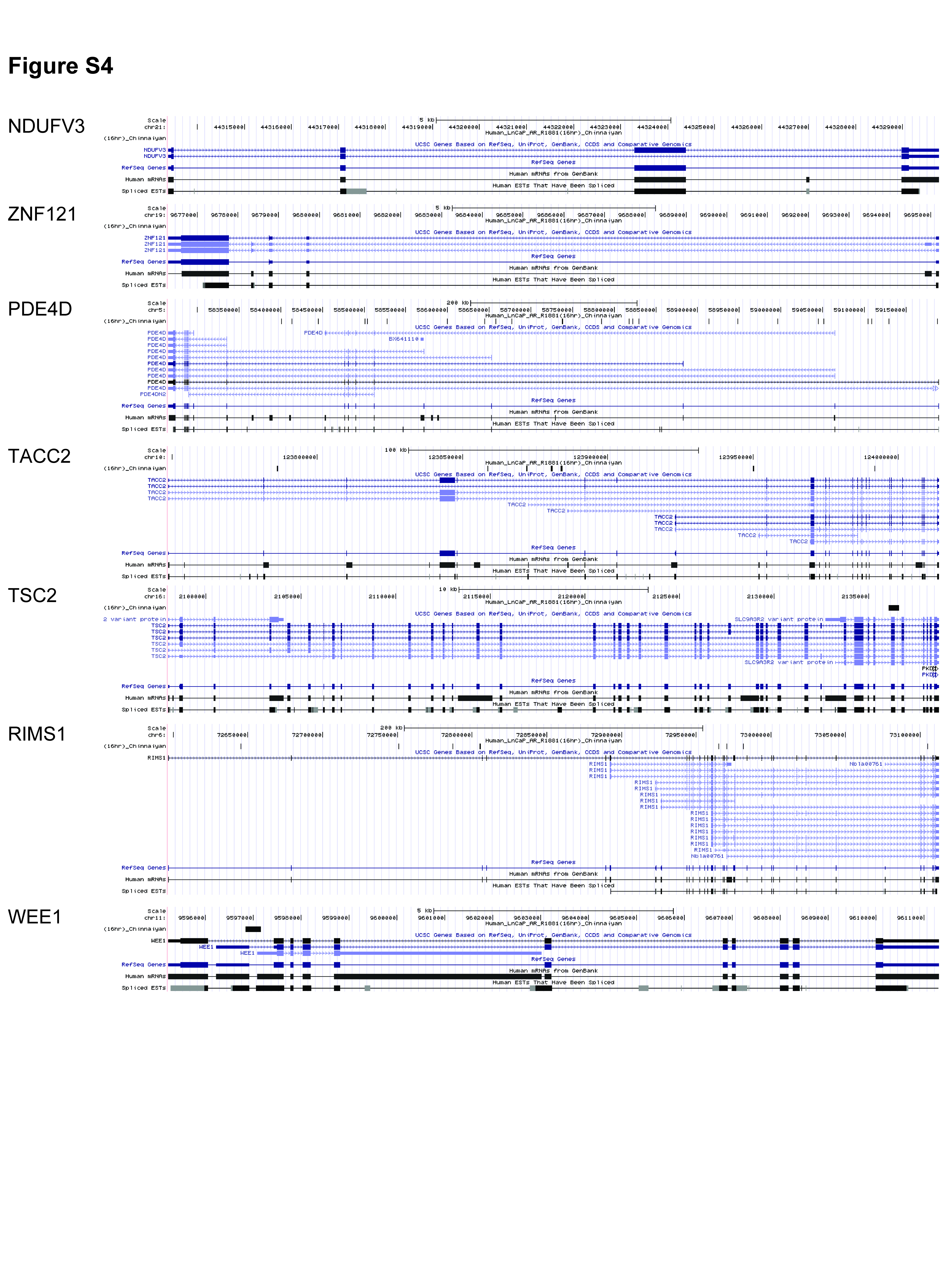

Supplement: Figure S4 — Expression of novel mRNA isoforms is both directly and indirectly regulated by androgens. (A) UCSC Genome Browser showing chromosomal locations and annotated transcript details for each novel androgen-regulated alternative mRNA isoform. Custom tracks (black bars below “Human_LNCaP_AR_R1881(16h)_Chinnaiyan”) for each gene show locations of AR binding site(s) within the LNCaP genome as determined by ChIP-Seq [34]. (TIF) [file pone.0029088.s005.tif]
