# Supplementary material for: Identification of Novel Androgen-Regulated Pathways and mRNA Isoforms through Genome-Wide Exon-Specific Profiling of the LNCaP Transcriptome
Source: PLoS One. 2011 Dec 14;6(12):e29088. doi: 10.1371/journal.pone.0029088 (PMC3237596; doi:10.1371/journal.pone.0029088)
Supplement: Table S2 — Overlap of identified androgen-regulated genes with previously-published datasets. (DOC) [file pone.0029088.s007.doc]

**Table S2**

| **Study** | **Technology** | **Dataset** | **Number of hits** | **% overlap**  **(n= )** |
| --- | --- | --- | --- | --- |
| (Li et al. 2008) | mRNA-Seq | Androgen-regulated gene and alternative mRNA isoform expression | 687 | 9.6 (53) |
| (Nelson et al. 2002) | cDNA microarrays on 20,000 genes | Androgen-regulated gene expression | 146 | 9.8 (54) |
| (Velasco et al. 2004) | Oligo-nucleotide array | Androgen-regulated gene expression | 692 | 7.6 (42) |
| (Segawa et al. 2002) | Affymetrix HuGene FL oligonucleotide arrays | Androgen-regulated gene expression | 525 | 8.4 (46) |
| (Xu et al. 2001) | Serial Analysis of Gene Expression (SAGE) | Androgen-regulated gene expression | 57 | 1 (6) |
| (DePrimo et al. 2002) | DNA Microarray ~18000 genes. | Androgen-regulated gene expression | 439 | 18.9 (104) |
| (Ngan et al. 2009) | ABI Human Genome Survey Microarray V2.0 | Androgen-regulated gene expression | 619 | 231 (42) |
