# Supplementary material for: Identification of Novel Androgen-Regulated Pathways and mRNA Isoforms through Genome-Wide Exon-Specific Profiling of the LNCaP Transcriptome
Source: PLoS One. 2011 Dec 14;6(12):e29088. doi: 10.1371/journal.pone.0029088 (PMC3237596; doi:10.1371/journal.pone.0029088)
Supplement: Table S3 — Genomic sequence of oligonucleotide primers. (A) qPCR for gene expression analysis (B) RT-PCR for splicing analysis (C) RT-PCR for gene expression analysis (D) qPCR for ChIP analysis. Where possible, primers were designed so that amplicons spanned exon junctions. Where shown predicted amplicon sizes was obtained using the in silico PCR program http://genome.ucsc.edu/index.html?org=Human&db=hg18&hgsid=142437216. (DOC) [file pone.0029088.s008.doc]

**Table S3**

|  | **Gene name** | **Primer position** | **Sequence (5’ → 3’)** | **Predicted PCR product sizes (bp)** |
| --- | --- | --- | --- | --- |
| **(A)** | *KLK3* | Forward | ATGTGGGTCCCGGTTGTCT |  |
|  |  | Reverse | AGCGCCAATCCACGTCA |  |
|  | *GAPDH* | Forward | AACGACCACTTTGTCAAGCTCAT |  |
|  |  | Reverse | CTTACTCCTTGGAGGCCATGT |  |
| **(B)** | *PDE4D* | Exon 2 (forward) | TTGGAACAAGCTGACTTGAAAA | 205; 314 |
|  |  | Exon 3 (forward) | TAATCCGCATTGTCCAAACC |  |
|  |  | Exon 7 (reverse) | TGTGGACAAAATTTGCTTGG |  |
|  | *TSC2* | Exon 8 (forward) | GCCGTGGTCTGCTACAACTG | 290; 339 |
|  |  | Exon 10 (reverse) | AACACAGATGTCGGCGAGTT |  |
|  |  | Exon 41 (forward) | GCCTCACAGGTGCATCATAG |  |
|  |  | Exon 42 (reverse) | GCACTGGGGTCAGGACTTTA |  |
|  | *ZNF121* | Exon 4 (forward) | AAACCAAAGTGTCAGCACCTC | 150; 208 |
|  |  | Exon 6 (reverse) | TCCAGAGATGACTGGGAAGC |  |
|  | *TACC2* | Exon 12 (forward) | CACTGAGGGAAGACGAGAGG | 229 |
|  |  | Exon 12 (reverse) | TGCTGCTTTCCAGAAGGAAT |  |
|  |  | Exon 14 (forward) | TTGTCCCGGGAAACTTTG | 115 |
|  |  | Exon 14 (reverse) | AGTCGCAACTGCCAAAGAG |  |
|  |  | Exon 15 (forward) | ATAAAAAGAGGCCCCACAGC | 112 |
|  |  | Exon 15 (reverse | CCAGCAGGTTCAGTGTCTCA |  |
|  |  | Exon 21 (forward) | GCCAGATGAAGAGAGCCTTG | 171 |
|  |  | Exon 21 (reverse) | ACCCCTTCTGCACTCTCTGA |  |
|  | *RIMS1* | Exon 8 (forward) | CATCCTGTAACGTGGCAACC | 200; 301 |
|  |  | Exon 9 (reverse) | TGTCCAACTACATCTGCTAGGC |  |
|  |  | Exon 34 (forward) | TGGAAAATGGGGCCTGTAT |  |
|  |  | Exon 35 (reverse) | TCCAGAGATGACTGGGAAGC |  |
|  | *WEE1* | Exon 1 (forward) | AAGCTGCGACTCTTCGACAC | 212; 320 |
|  |  | Exon 2 (forward) | GGTGTTCAGCACCTGTGTTTT |  |
|  |  | Exon 3 (reverse) | CACTGTCCTGAGGAATGAAGC |  |
|  | *NDUFV3* | Exon 2 (forward) | GCCACAGAATTCCAAGAAGC | 196; 299 |
|  |  | Exon 3 (forward) | CACCAAGCAATTTGGAGACA |  |
|  |  | Exon 4 (reverse) | CCCTCAGTGTCGAGGTGACT |  |
| **(C)** | *mTOR* | Forward | GCACATTGACTTTGGGGACT |  |
|  |  | Reverse | ACTGGCCAGCAGAGTAGGAA |  |
|  | *DDIT4* | Forward | CTGTCCTCACCATGCCTAGC |  |
|  |  | Reverse | TGGCACACAAGTGTTCATCC |  |
|  | *DEPDC6* | Forward | AAGCTGATGAGCCCTGAAAA |  |
|  |  | Reverse | TTGTTGGACACATGCTGGAT |  |
|  | *TULP4* | Forward | GTGAGGTGGAATGAGCCCTA |  |
|  |  | Reverse | GTTGGTCGTCAGGAGTCCAT |  |
|  | *KITLG* | Forward | GAAGCAGGGACAGTGGAGAG |  |
|  |  | Reverse | TTGGAAGATTTGCCACCAAT |  |
|  | *FZD5* | Forward | ACGGGTACCCAGCCTGTC |  |
|  |  | Reverse | AACCTGTTGGTTGCTTTTTCC |  |
|  | *CYTH1* | Forward | GAAGGGCTCAACAAGACAGC |  |
|  |  | Reverse | CATCATCCGGTCGATCTTCT |  |
|  | *TLR* | Forward | GTGCCAGAAACTTCCCATGT |  |
|  |  | Reverse | TCCAGCTGAACCTGAGTTCC |  |
|  | *PI3KR1* | Forward | TTTGACTCTCCCGGATCTTG |  |
|  |  | Reverse | TTCACTGTAAACGGCTGCTG |  |
|  | *SH3RF1* | Forward | GCAGACAAAGATTGCCTTCC |  |
|  |  | Reverse | TGAGGGAAGTGAAGGAGTGC |  |
|  | *DDX58* | Forward | GCTTTGCCTGCTATGAAAGG |  |
|  |  | Reverse | TCTCAGCTGTTGCTCCAGAA |  |
| **(D)** | *DP1* | Forward | AATGTCTTTGGCTTGGCCAG |  |
|  |  | Reverse | GCCACAAAAGGGACCAAGTCA |  |
|  | *DP2* | Forward | TCCTCTCCCCCTGTCCTGA |  |
|  |  | Reverse | GAGGTCCCAAAGCAGCATTTC |  |
|  | *GREB1* | Forward | TCTGTGGAGTGCCTGAAGTG |  |
|  |  | Reverse | GCCAATGCTTTGCCATTATT |  |
